# The Trade-Off Between Sterility and Structural Integrity in Sterilized Alginate Hydrogels

**DOI:** 10.3390/gels12060478

**Published:** 2026-05-29

**Authors:** Paula Kaufelde, Anete Vircava, Ingus Skadiņš, Kristiāna Rubeze, Jevgenijs Proskurins, Konstantīns Logviss, Agnese Brangule

**Affiliations:** 1 Laboratory of Finished Dosage Forms, Faculty of Pharmacy, Riga Stradiņš University, LV-1007 Riga, Latvia; 2 Baltic Biomaterials Centre of Excellence, Headquarters at Riga Technical University, LV-1048 Riga, Latvia; 3 Department of Pharmacology and Pharmacotherapy, Riga Stradiņš University, LV-1007 Riga, Latvia; 4 Department of Biology and Microbiology, Riga Stradiņš University, LV-1007 Riga, Latvia; 5 Department of Physics, Riga Stradiņš University, LV-1067 Riga, Latvia

**Keywords:** hydrogel sterilization, microbial inactivation, structural integrity, rheology, UV irradiation, ethylene oxide, gamma irradiation, steam sterilization, high hydrostatic pressure, alginate model system

## Abstract

Sterilization is essential for hydrogel-based biomaterials, but it can also determine the final material state. This study used ionically crosslinked alginate hydrogels as a model system to evaluate sterilization as a coupled process linking microbial inactivation and hydrogel structural reorganization. Steam sterilization, gamma irradiation, ethylene oxide (EtO), ultraviolet (UV) irradiation, and high hydrostatic pressure (HHP) treatment were assessed within the same model system. Microbiological effectiveness was assessed using surface- and matrix-associated contamination models, while structural responses were evaluated by rheology, dimensional changes, and swelling behavior. Steam sterilization, gamma irradiation, EtO, and selected HHP conditions resulted in no detectable microbial growth under the tested conditions, whereas UV irradiation was insufficient to eliminate detectable growth from matrix-associated contamination. However, microbiologically effective treatments produced distinct material profiles. Steam generated a compact and stiff hydrogel state, gamma irradiation produced softened but deformation-tolerant networks, EtO caused pronounced dimensional alteration and high deformability, and HHP produced softened, water-accessible hydrogels with parameter-dependent responses. These findings show that sterilization method selection should integrate microbial inactivation with the final structural state required for application-specific hydrogel performance.

## 1. Introduction

Sterilization of hydrogel-based biomaterials is a critical step in their translation from laboratory formulations to biomedical products [1,2]. Hydrogels are three-dimensional crosslinked polymer networks with high water content, tunable mechanical properties, flexibility, and the ability to incorporate small molecules, macromolecules, or bioactive compounds [3,4,5]. These properties make hydrogels relevant for a broad range of biomedical applications, including wound dressings, drug delivery systems, tissue engineering scaffolds, and soft biomedical devices [6,7].

Despite the extensive research on hydrogel synthesis and modification, with more than 15,000 publications reported annually in this field, sterilization remains comparatively underexplored as a factor that can determine the final hydrogel state [2,8]. Sterilization is often treated mainly as a technical post-processing step, whereas its influence on material behavior and application suitability is only partially considered.

For clinical applications, hydrogels must be sterilized to ensure microbiological safety. At the same time, sterilization methods expose materials to heat, radiation, chemical agents, surface irradiation, pressure, dehydration, or changes in the surrounding environment. These conditions may result in different post-sterilization material states rather than simply preserving or damaging the original hydrogel [9].

Although previous studies have shown that sterilization can alter hydrogel mechanical properties, swelling, degradation, printability, biocompatibility, or polymer structure, microbiological effectiveness and material response are often evaluated separately. This limits the ability to select sterilization methods based on the specific requirements of the final hydrogel application [8,10].

From a microbiological perspective, sterilization methods inactivate microorganisms through different biological effects, including DNA damage, protein denaturation, membrane disruption, oxidative stress, metabolic inactivation, and loss of essential cellular functions [11,12,13,14,15]. The response to sterilization may also depend on the type of microorganism, since Gram-positive bacteria, Gram-negative bacteria, and fungi differ in cell wall structure and stress resistance. These differences are especially relevant for hydrogel systems, where microorganisms may be located not only on the surface but also within or near the hydrated matrix [16,17,18].

The same sterilization methods can transform the hydrogel network through method-specific physicochemical effects. Gamma irradiation may induce polymer chain scission, radical formation, and secondary crosslinking, thereby altering viscoelastic behavior and network integrity. Steam sterilization may promote dehydration, polymer densification, and changes in the organization of ionic crosslinking. Ultraviolet irradiation is mainly surface-limited and may produce heterogeneous structural effects due to limited penetration, whereas ethylene oxide sterilization is suitable for heat-sensitive materials but requires aeration, residual control, and validation of material compatibility [19,20,21,22,23,24,25]. Therefore, similar microbiological outcomes can correspond to very different structural and functional hydrogel states.

As a result, a gap remains in understanding sterilization as a coupled process linking microbial inactivation, material transformation, and application-relevant performance. The key question is not only whether a method eliminates detectable microorganisms, but what kind of hydrogel state it produces and whether that state is suitable for the intended use.

In the present study, ionically crosslinked alginate hydrogels were selected as a model system. Alginate hydrogels are widely used in biomedical research due to their biocompatibility, mild gelation conditions, high water content, and relevance for wound care, tissue engineering, and drug delivery. Their structure is stabilized mainly by reversible ionic interactions between alginate chains and divalent cations, making them highly sensitive to external physicochemical stresses and suitable for evaluating how sterilization methods simultaneously affect microbial inactivation, rheological behavior, dimensional changes, and swelling capacity [26,27,28,29,30].

Previous studies on alginate-based materials have reported changes after autoclaving, UV irradiation, ethanol treatment, gamma irradiation, and ethylene oxide sterilization, including effects on polymer structure, swelling, mechanical behavior, printability, degradation, and biocompatibility. However, these effects have mostly been interpreted as individual material changes, rather than as part of an integrated microbiology–structure–application assessment [8,10,31,32].

To address this gap, the present study assessed steam sterilization, gamma irradiation, ethylene oxide sterilization, ultraviolet irradiation, and high hydrostatic pressure treatment (HHP) within the same alginate hydrogel model system. HHP was included as a non-thermal, pressure-based approach that applies uniform isostatic pressure at moderate temperatures and is widely used in the food industry for microbial inactivation in liquid and gel-like systems [33,34,35,36]. However, its use for soft biomedical hydrogels remains relatively underexplored. Including HHP enabled the evaluation of heat-, radiation-, chemical-, surface-, and pressure-based sterilization stresses within the same alginate hydrogel model system.

The aim of this study was to use ionically crosslinked alginate hydrogels as a model system to examine sterilization as a coupled process linking microbial inactivation, physicochemical stress, and hydrogel structural reorganization. Microbiological effectiveness was evaluated using both surface- and matrix-associated contamination models. Structural responses were assessed using rheological measurements, including storage modulus G′, loss factor tan δ, and the linear viscoelastic region, as indicators of stiffness, viscoelastic balance, deformation tolerance, and network integrity. Macroscopic geometry and swelling behavior were included as complementary indicators of dimensional stability and polymer–water interactions.

The objectives of this study were:

(i) to evaluate the microbiological effectiveness of different sterilization methods, including HHP, in contaminated alginate hydrogel model systems;
(ii) to determine how these sterilization methods affect the rheological, dimensional, and swelling properties of alginate hydrogel model systems; and
(iii) to integrate microbiological and structural data to identify application-relevant sterilization trade-offs in alginate hydrogel model systems.

## 2. Results and Discussion

### 2.1. Microbiological Effectiveness of Sterilization Methods

Sterilization effectiveness was assessed using two contamination approaches. Gram-negative bacteria (*E. coli*, ESBL, and *P. aeruginosa*) were incorporated into the alginate matrix before gelation, whereas Gram-positive bacteria (*S. aureus*, MRSA) and *C. albicans* were introduced by post-gelation immersion of pre-formed hydrogel slices, as described in Section 4.3.

EtO treatment, steam sterilization, and gamma irradiation resulted in no detectable microbial growth in either surface-exposed or internally sampled regions, regardless of microorganism type or contamination mode. This indicates that, under the tested conditions, these treatments were effective not only at the hydrogel surface but also within the internal matrix.

**Figure 1:**
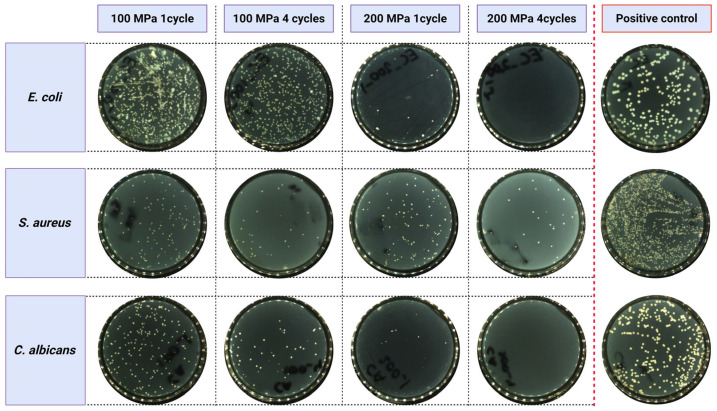
Representative agar plates showing microbial survival after HHP treatment in an alginate hydrogel model system at different pressure levels and cycle numbers. Created in BioRender. Brangule, A. (2026) https://BioRender.com/d4e264p.

HHP treatment showed a clear dependence on pressure level and the number of cycles (Figure 1). Treatment at 100 MPa for 10 min, applied either as one or four cycles, did not result in an observable reduction in microbial growth. Microbial growth remained comparable to untreated positive controls, indicating that 100 MPa is below the inactivation threshold for the tested microorganisms under these conditions.

At 200 MPa/one cycle reduced detectable growth of Gram-negative bacteria (*E. coli*, ESBL) and *C. albicans* was observed. After 4 cycles at 200 MPa, no detectable growth was observed for these microorganisms. This observation is consistent with our previous study on *E. coli* in suspension (10^6^ CFU/mL), where 200 MPa was applied for 4 cycles, resulting in no detectable viable cells and an effect comparable to a single 300 MPa cycle [37]. Together, these findings demonstrate that HHP effectiveness depends on both the applied pressure and the number of cycles, rather than pressure alone.

In contrast, Gram-positive *S. aureus* exhibited higher baroresistance. Our preliminary experiments with pure *S. aureus* and MRSA suspensions (10^6^ CFU/mL) demonstrated that absence of detectable growth required pressures ≥ 500 MPa, consistent with literature reports of greater baroresistance in Gram-positive bacteria compared with Gram-negative strains [38,39,40]. However, in the present hydrogel system, after post-gelation immersion with *S. aureus*, reduced detectable growth was already observed after four cycles at 200 MPa, while no detectable growth was observed after four cycles at 300 MPa.

This difference may be related to the hydrogel-associated experimental environment, as HHP inactivation of *S. aureus* depends on both microorganism-related factors and process-related parameters, including strain, growth phase, pressure level, treatment mode, and the composition of the surrounding matrix [41,42,43,44]. In the present study, *S. aureus* and MRSA were introduced by immersing pre-formed Ca-alginate hydrogel slices in bacterial or fungi suspensions; therefore, HHP was applied to hydrogel-associated cells rather than to free planktonic suspensions. Accordingly, the observed response should be interpreted as the combined effect of microbial pressure resistance, repeated pressure cycling, and the hydrogel-based matrix [41,42].

**Figure A1:**
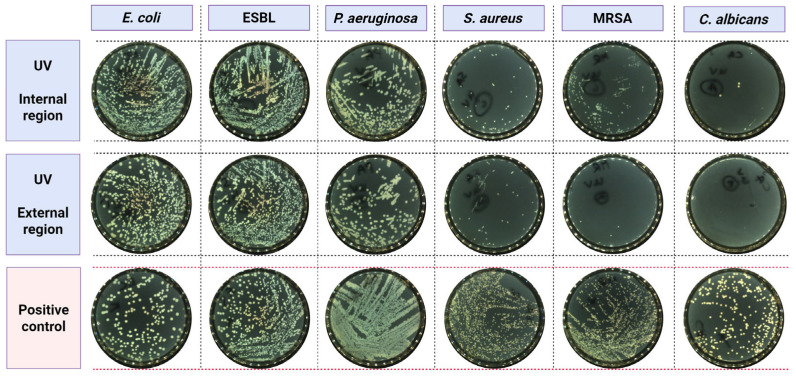
Microbial recovery after UV irradiation in the hydrogel model. Created in BioRender. Brangule, A. (2026) https://BioRender.com/jtmppjp.

UV irradiation showed limited microbiological effectiveness in the alginate hydrogel system (Appendix A.1, Figure A1). Gram-negative bacteria (*E. coli*, ESBL-producing *E. coli*, and *P. aeruginosa*), incorporated into the alginate matrix before gelation remained detectable after UV treatment, with detectable growth observed in both external surface and internal cross-sectional samples. For *S. aureus*, MRSA, and *C. albicans*, introduced by post-gelation immersion, UV treatment reduced visible microbial growth compared with untreated positive controls, but did not eliminate detectable growth. Residual growth was still observed, particularly for *S. aureus* and MRSA, indicating that even surface-associated contamination was not fully eliminated under the applied conditions.

The limited effect of UV treatment can be explained by the limited penetration of UV-C radiation into thicker or non-uniform hydrated polymer systems and by shielding effects at the hydrogel surface [8,18]. In matrix-contaminated samples, microorganisms located within the alginate network are not directly exposed to the incident UV radiation. In immersion-contaminated samples, bacteria may still be protected by surface irregularities, local aggregation, or partial penetration into the near-surface hydrogel region [45,46]. Therefore, the applied UV dose should be interpreted mainly as a surface treatment, and under the present conditions, it was insufficient to reliably eliminate detectable microorganisms from contaminated alginate hydrogels.

These findings show that microbiological effectiveness in alginate hydrogels depends not only on the sterilization method itself, but also on the treatment’s ability to reach microorganisms within the hydrated three-dimensional matrix. EtO treatment, steam sterilization, gamma irradiation, and selected HHP conditions resulted in no detectable microbial growth under the tested conditions. In contrast, UV irradiation showed limited effectiveness and did not reliably eliminate detectable microorganisms from contaminated alginate hydrogels.

These microbiological results show that hydrogel sterilization cannot be evaluated only as a surface decontamination process, especially when contamination may occur within or close to the hydrogel matrix. They also provide the basis for interpreting how microbiologically effective and ineffective treatments affect hydrogel structure, swelling behavior, and rheological properties in the following sections.

### 2.2. Rheological Characterization of Sterilization-Induced Mechanical States in Alginate Hydrogels

Rheological analysis was used as the primary tool to characterize how sterilization changed the functional mechanical state of alginate hydrogels.

The unsterilized hydrogel served as the reference state for defining sterilization-induced shifts in stiffness, viscoelastic balance, and deformation tolerance. This approach enabled the treatments to be interpreted as structure-setting processes that generated distinct post-sterilization mechanical states.

**Figure 2:**
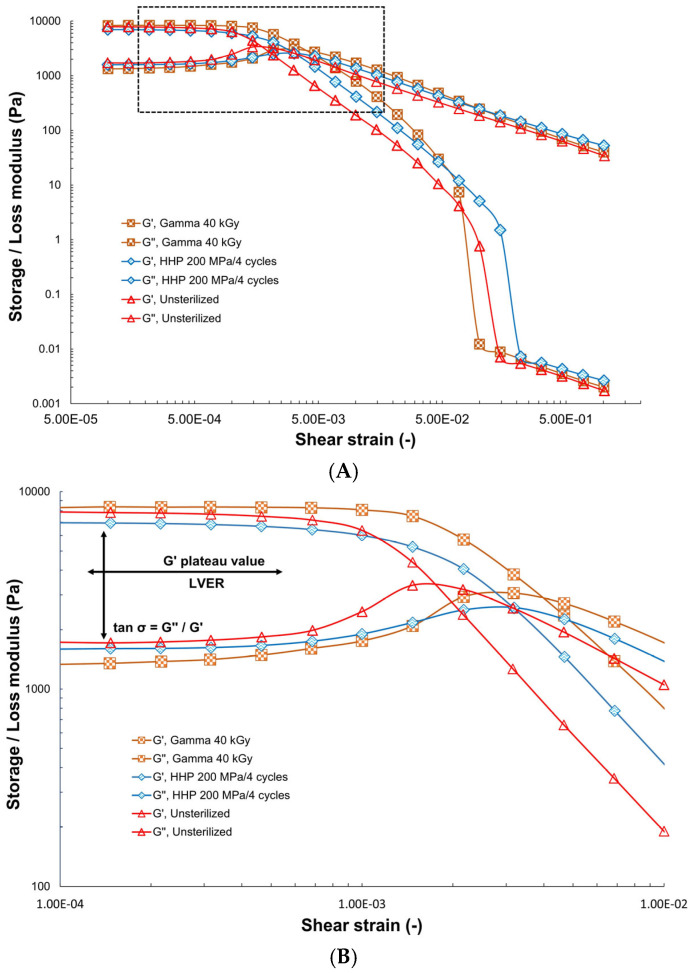
Representative amplitude sweep curves and rheological parameter extraction. (**A**) Storage modulus (G′) and loss modulus (G″) as a function of shear strain (γ) for representative alginate hydrogel samples: unsterilized hydrogel, HHP-treated hydrogel at 200 MPa/4 cycles, and gamma-irradiated hydrogel at 40 kGy. The dashed rectangle indicates the low-strain region used for extracting rheological parameters. (**B**) Enlarged view of the highlighted linear viscoelastic region (LVER), showing the plateau G′, the LVER limit, and the loss factor, defined as tan δ = G″/G′.

The rheological response was characterized using three complementary parameters obtained from amplitude sweep experiments (Figure 2): the plateau storage modulus (G′), the linear viscoelastic region (LVER), and the loss factor (tan δ = G″/G′). Together, these parameters describe hydrogel stiffness (G′), deformation tolerance within the linear viscoelastic region (LVER), and the balance between elastic and viscous behavior (tan δ). Their combined interpretation enabled the definition of sterilization-induced changes at the level of functional network response [47,48].

#### 2.2.1. Rheological Profile of the Unsterilized Reference Hydrogel

The unsterilized hydrogel defined the reference rheological profile of the Ca-alginate network before sterilization. In the highlighted LVER region of Figure 2B, the reference hydrogel exhibited a plateau storage modulus G′ of 14.1 ± 2.3 kPa, indicating a stable elastic response under the applied measurement conditions. The LVER extended up to approximately 0.15 ± 0.06% strain, after which the material response deviated from the linear viscoelastic regime. The loss factor tan δ was 0.30 ± 0.06, indicating predominantly elastic behavior with a measurable viscous contribution.

This profile represents a predominantly elastic hydrogel state with a storage modulus in the kilopascal range and limited deformation tolerance. It was used as the baseline for comparing how each sterilization method shifted the balance between stiffness, elasticity, and deformation tolerance in the following sections.

#### 2.2.2. Sterilization-Induced Shifts in Rheological Parameters

Sterilization-induced rheological changes were evaluated using the plateau G′, LVER, and tan δ values of the treated hydrogels relative to the unsterilized reference profile defined in Section 2.2.1. Figure 2 shows representative amplitude sweep profiles for the unsterilized hydrogel, gamma-irradiated hydrogel at 40 kGy, and HHP-treated hydrogel at 200 MPa/4 cycles, while the complete rheological profiles for all treatment groups are available in the Zenodo dataset listed in the Data Availability Statement.

Because LVER showed higher replicate-to-replicate variability than G′ and tan δ, it was interpreted as a supporting deformation-tolerance parameter, whereas statistical analysis was mainly used to support treatment-dependent shifts in G′ and tan δ.

**Figure 3:**
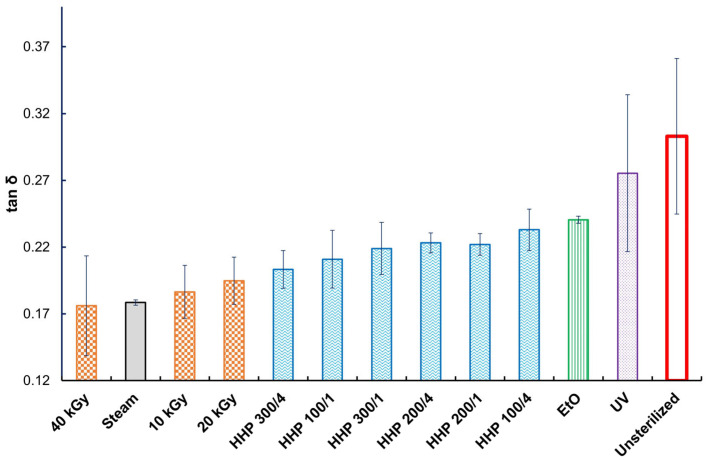
Loss factor of unsterilized and sterilized alginate hydrogels. Loss factor values (tan δ = G″/G′) derived from amplitude sweep measurements for unsterilized, steam-sterilized, gamma-irradiated, HHP-treated, EtO-treated, and UV-treated hydrogels. Lower tan δ values indicate a more elastic, solid-like response, whereas higher values indicate a greater viscous contribution. Data are presented as mean ± standard deviation, *n* = 3. Statistical comparisons were performed using one-way ANOVA followed by Tukey’s post hoc test.

Figure 3 summarizes the corresponding changes in tan δ across all treatment groups. One-way ANOVA indicated significant treatment-dependent differences in tan δ values (*p* < 0.001), supporting the interpretation that sterilization produced method-dependent shifts in the viscoelastic balance of the hydrogels.

Steam sterilization produced the highest stiffness among the tested methods, with G′ increasing to 22.5 ± 1.2 kPa. This was accompanied by a low tan δ value of 0.18 ± 0.02, indicating a predominantly elastic response. The LVER of steam-sterilized hydrogels was 0.31 ± 0.32%, indicating high variability in the linear viscoelastic range. Therefore, steam-treated samples were interpreted primarily as a stiffened hydrogel state, while the LVER response was considered variable.

In contrast, gamma irradiation produced dose-related softening while maintaining elastic dominance. At 10 and 20 kGy, G′ decreased to 8.5 ± 0.7 and 8.4 ± 1.3 kPa, respectively, and further decreased to 6.9 ± 1.2 kPa at 40 kGy. Despite this reduction in stiffness, tan δ remained low across all gamma-treated groups, ranging from 0.18 ± 0.04 to 0.19 ± 0.02. Mean LVER values in gamma-irradiated hydrogels were approximately 0.28–0.33%, generally above the unsterilized reference. Together with reduced G′ and low tan δ, this supports the interpretation of a softened, predominantly elastic hydrogel state.

EtO sterilization followed a similar softening direction, but with a distinct deformation profile. G′ decreased to 7.5 ± 0.9 kPa, while tan δ reached 0.24 ± 0.02, indicating predominantly elastic behavior with a greater viscous contribution than in steam- and gamma-treated samples. Notably, EtO-treated hydrogels also showed the broadest LVER among all treatments, 0.83 ± 0.31%, supporting the interpretation of a soft but highly deformation-tolerant state.

UV treatment largely preserved apparent stiffness but strongly restricted the linear deformation range. UV-treated hydrogels showed a G′ of 16.3 ± 1.3 kPa and a tan δ of 0.28 ± 0.06, both values close to the unsterilized reference. However, the LVER was low at 0.03 ± 0.02%, indicating that preserved stiffness was accompanied by a markedly reduced strain range for stable linear viscoelastic response.

HHP treatment produced another distinct response, characterized mainly by softening across all tested pressure and cycle combinations. G′ ranged from 4.7 ± 1.1 kPa for 300 MPa/4 cycles to 9.1 ± 5.1 kPa for 200 MPa/1 cycle. Across HHP conditions, tan δ remained within a relatively narrow range of 0.20 ± 0.01 to 0.23 ± 0.02, indicating that the gels retained predominantly elastic behavior despite reduced stiffness. LVER values were generally limited and variable, ranging from 0.03 ± 0.02% to 0.17 ± 0.20%, with the highest value observed at 300 MPa/4 cycles. Within experimental variability, one- and four-cycle treatments at the same pressure showed broadly comparable rheological profiles, indicating that cycle number did not produce a clearly proportional change in the material response.

Taken together, the sterilization methods produced distinct rheological shifts rather than a uniform mechanical response. Steam increased stiffness, while its LVER response remained variable; gamma irradiation produced dose-related softening while maintaining elastic behavior; EtO generated a soft and highly deformation-tolerant state; UV preserved apparent stiffness but narrowed the LVER; and HHP produced softened states with broadly comparable tan δ values across pressure–cycle combinations. These method-specific parameter shifts provide the basis for mapping the treated hydrogels into post-sterilization mechanical regions in the following section.

#### 2.2.3. G′–LVER Mapping of Rheological Profiles

**Figure 4:**
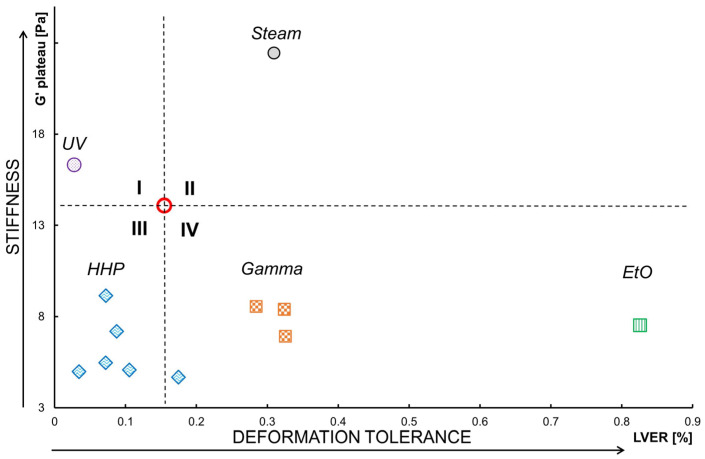
Comparative G′–LVER map and rheological classification of sterilized Ca-alginate model hydrogels. The plateau storage modulus (G′) and the linear viscoelastic region (LVER) were used to map hydrogel stiffness and deformation tolerance. Dashed lines indicate the unsterilized reference values used to define four operational regions: (I) stiff–low LVER, (II) stiff–high LVER, (III) soft–low LVER, and (IV) soft–high LVER. The map is based on mean values and is interpreted as an operational comparison of rheological profiles within this alginate model system.

Based on the rheological parameters defined above, hydrogel stiffness and deformation tolerance were compared using a G′–LVER map (Figure 4). One-way ANOVA indicated significant treatment-dependent differences in both G′ values (*p* < 0.001) and LVER values (*p* < 0.01), supporting the use of these parameters for comparative mapping of post-sterilization rheological profiles.

The unsterilized hydrogel was used as the reference point for defining the threshold values of the map. Its plateau storage modulus, G′ = 14.1 ± 2.3 kPa, was used to distinguish hydrogels that were softer or stiffer than the unsterilized reference. Similarly, the reference LVER value of 0.15 ± 0.06% was used to distinguish hydrogels with lower or higher deformation tolerance than the reference. Based on these thresholds, four regions were defined: (I) stiff–low LVER, (II) stiff–high LVER, (III) soft–low LVER, and (IV) soft–high LVER. These regions should be interpreted as relative rheological profiles within the present alginate model system, rather than as universal material classes.

UV-treated hydrogels were located in Region I. Their G′ was higher than that of the unsterilized reference, 16.3 ± 1.3 kPa, but their LVER was clearly lower, 0.03 ± 0.02%. Thus, UV treatment preserved apparent stiffness but reduced the strain range over which the hydrogel maintained a stable viscoelastic response.

Steam-sterilized hydrogels showed the opposite trend and were located in Region II. They combined the highest G′ value, 22.5 ± 1.2 kPa, with an LVER above that of the reference hydrogel, 0.31 ± 0.32%. This indicates a stiff profile with a higher, although variable, LVER.

Gamma-irradiated hydrogels were located in Region IV. Their G′ values were lower than those of the unsterilized reference, ranging from 6.9 to 8.5 kPa, while their LVER values were higher, ranging from 0.28 to 0.33%. The 10, 20, and 40 kGy samples formed a compact group in this region, with 40 kGy showing the lowest stiffness. This indicates that gamma irradiation softened the hydrogels while increasing their tolerance to deformation within the LVER.

EtO-treated hydrogels were also located in Region IV. Their G′ was similar to the gamma-treated samples, 7.5 ± 0.9 kPa, but their LVER was much higher, 0.83 ± 0.31%. Therefore, EtO produced a soft hydrogel with the highest deformation tolerance among the tested treatments.

Most HHP-treated hydrogels were located in Region III. Their G′ values were below the unsterilized reference, ranging from 4.7 to 9.1 kPa, and their LVER values were generally low, ranging from 0.03 to 0.17%. This indicates that HHP mainly produced soft hydrogels with limited deformation tolerance. The 300 MPa/4 cycle condition was closest to the boundary with Region IV, suggesting a shift toward greater deformation tolerance at the highest pressure and cycle combination.

Taken together, the G′–LVER classification shows that sterilization methods produced different rheological profiles rather than a uniform shift from the unsterilized hydrogel. Steam produced a stiff–high LVER profile, gamma irradiation and EtO produced soft–high LVER profiles, UV produced a stiff–low LVER profile, and HHP produced mainly soft–low LVER profiles. This classification supports comparison of the final hydrogel profiles by combining stiffness and deformation tolerance rather than interpreting G′ or LVER separately.

### 2.3. Dimensional and Swelling Responses as Supporting Bulk-Level Indicators

**Figure 5:**
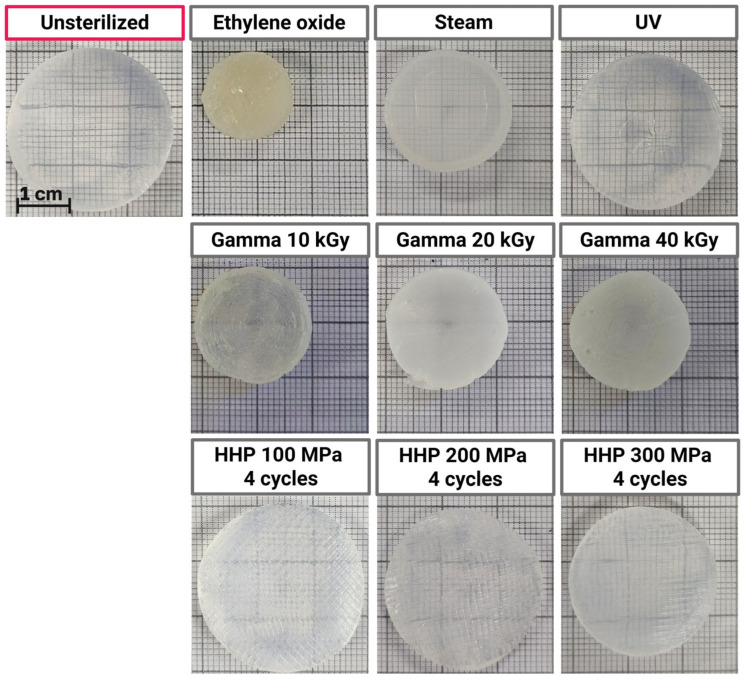
Sterilization method-dependent dimensional changes in sterilized Ca-alginate model hydrogels. Representative samples are shown for the unsterilized hydrogel and hydrogels treated with ethylene oxide, steam, UV irradiation, gamma irradiation at 10, 20, and 40 kGy, and HHP at 100–300 MPa for 4 cycles. Scale bar = 1 cm. Created in BioRender. Brangule, A. (2026) https://BioRender.com/4t1hyhd.

Macroscopic geometry and swelling behavior were used as complementary indicators at the bulk level. The data supported the interpretation that sterilization produced treatment-dependent dimensional and swelling responses. These measurements complemented the rheological classification by showing whether changes in stiffness and LVER were accompanied by changes in sample dimensions and rehydration capacity. Representative hydrogel appearances after sterilization are shown in Figure 5.

Sterilization produced method-dependent dimensional changes. EtO caused the strongest macroscopic alteration, with mass reduced by approximately 80%, height by 32%, and diameter by 40%. Steam sterilization also caused substantial shrinkage, with approximately 55% mass loss and about 23% reductions in both height and diameter, consistent with the compact, stiff rheological profile observed after steam treatment. Gamma irradiation produced dose-dependent dimensional changes, with mass loss increasing from approximately 50% at 10–20 kGy to approximately 65% at 40 kGy. HHP-treated hydrogels showed pressure-dependent reductions in mass and height, reaching about 55–60% at 300 MPa, while diameter changes remained limited, generally within ±7%. UV-treated hydrogels showed the smallest dimensional changes, with approximately 12% mass loss and no major changes in height or diameter.

Swelling behavior further differentiated the sterilized hydrogel states. Drying removed a similar fraction of water from all samples, typically 93–95% of the post-sterilization mass. Differences between treatments became more evident during rehydration. In deionized water, swelling remained limited for all groups, whereas PBS induced greater expansion, consistent with the ion-sensitive nature of Ca-alginate hydrogels. The unsterilized hydrogel showed the highest swelling capacity, reaching approximately 4500%, while all sterilized samples showed reduced rehydration capacity.

Among sterilized samples, HHP-treated hydrogels showed the highest swelling capacity, increasing with pressure from approximately 800–900% at 100 MPa to 1500–1900% at 300 MPa after 24 h in PBS. This supports the interpretation that HHP produced softened hydrogels with higher rehydration capacity, despite their generally low LVER. Gamma-irradiated hydrogels showed moderate swelling, with lower swelling at higher irradiation dose, consistent with dose-dependent changes in the bulk hydrogel state. Steam- and EtO-treated samples showed lower swelling of approximately 480–540%, supporting the interpretation that they are in more compact or strongly altered post-sterilization states.

Direct comparison with literature data is limited because sterilization responses depend not only on the sterilization method, but also on hydrogel synthesis parameters, including alginate type, polymer concentration, crosslinking conditions, geometry, hydration state, and whether sterilization is applied before or after gel formation [26,27,31,32]. Comparable studies on pre-formed hydrated Ca-alginate hydrogel slices are limited, as many reports use alginate beads, alginate solutions, or alginate-based composite systems [8,31,49]. However, several trends are consistent with previous reports. In pre-formed alginate-based hydrogels, Stoppel et al. reported that UV treatment resulted in limited changes in water retention and shear rheology, whereas autoclaving led to water loss and increased stiffness [8]. This agrees with the present results, in which UV-treated hydrogels largely preserved dimensions and showed G′ and tan δ values close to those of the unsterilized reference, whereas steam sterilization produced a compact, shrunken, and stiff profile. For gamma irradiation and EtO, fewer data are available for pre-formed hydrated Ca-alginate hydrogels. Leo et al. reported that gamma irradiation and EtO treatment reduced alginate viscosity and Ca-alginate gel strength and increased bead diameter, indicating a weaker gel-forming system [49]. This trend is consistent with the softening and dimensional changes observed after gamma irradiation and EtO treatment in the present study, although their system was not directly identical to the hydrogel slices used here. Comparable data for HHP-treated Ca-alginate hydrogels remain limited.

Taken together with the rheological data, the dimensional and swelling results supported the treatment-specific hydrogel profiles defined primarily by G′–LVER mapping. Steam combined high stiffness with shrinkage and moderate swelling, consistent with a compact post-sterilization profile. Gamma irradiation combined reduced stiffness, low tan δ, increased LVER, and dose-dependent dimensional changes, consistent with a softened but deformation-tolerant bulk hydrogel profile. EtO combined reduced stiffness with the strongest dimensional alteration and the highest LVER, consistent with a soft and highly deformation-tolerant post-sterilization state. HHP combined reduced stiffness with the highest swelling capacity among the sterilized samples, consistent with a softened and highly rehydratable post-sterilization state despite limited deformation tolerance in rheological testing. UV preserved macroscopic shape and apparent stiffness, but its very low LVER indicated reduced linear viscoelastic stability under strain. Thus, geometry and swelling provided complementary bulk-level evidence for the treatment-specific hydrogel profiles, which were defined primarily by rheology.

### 2.4. Microbiology–Structure Trade-Offs and Application-Oriented Interpretation

The integrated assessment of sterilization outcomes is summarized in Table 1. The table combines microbiological outcome, G′–LVER rheological profile, swelling response, dimensional response, cost, implementation burden, and application-oriented interpretation. This format was used to integrate sterilization methods as post-processing strategies that affect both microbial control and the final hydrogel state [50].

Steam sterilization, gamma irradiation, EtO, and selected HHP conditions resulted in no detectable microbial growth. UV irradiation was insufficient when microorganisms were present within the hydrogel matrix. However, the structurally relevant outcomes differed between methods. Steam produced a stiff–high LVER profile with reduced swelling and shrinkage. Gamma irradiation and EtO produced soft–high LVER profiles, but EtO caused the strongest dimensional alteration. HHP produced mainly soft–low LVER profiles with the highest swelling among sterilized samples. UV produced a stiff–low LVER profile and preserved dimensions most effectively, but did not reliably eliminate microorganisms inside the hydrogel.

These results show that microbial inactivation, dimensional preservation, swelling response, and rheological stability are separate outcomes. A method may be microbiologically effective while substantially altering the hydrogel state, or it may preserve the hydrogel’s visible geometry while failing to provide reliable sterilization. Therefore, the selection of the sterilization method should be based on the required post-sterilization hydrogel profile rather than on microbial outcomes alone.

Cost and implementation burden further differentiate the methods. Steam sterilization has a low practical burden and is widely available, but its use is limited by its heat- and moisture-sensitivity. Gamma irradiation is scalable and applicable to packaged products, but it requires access to irradiation facilities, dose validation, and material compatibility assessment. EtO is applicable to heat-sensitive products, but requires aeration, residual control, and regulatory validation [52]. HHP is scalable in industrial processing but requires specialized equipment, high initial investment, and pressure–cycle optimization [37,53]. UV irradiation is a low-cost surface treatment, but its limited penetration and sensitivity to shading restrict its use to directly exposed surfaces [8,18]. Thus, the most cost-effective method is not necessarily the lowest-cost process, but rather the one that provides acceptable microbial control while producing a suitable final hydrogel.

Application-oriented interpretation was based on the material profiles obtained in this study and on literature-supported requirements for hydrogel applications. Soft and deformation-tolerant profiles, such as those obtained after gamma irradiation or EtO treatment, may be relevant for soft terminally sterilized or conformable hydrogel formats, provided that dose effects, residual safety, dimensional stability, and biological performance are validated [60]. Highly water-accessible hydrogels, such as those produced by HHP, may be relevant for hydrated, thermally sensitive, or drug-loaded systems where swelling and network accessibility influence loading, diffusion, or release [58,61]. Compact and stiff hydrogels, such as those produced by steam sterilization, may be relevant where mechanical robustness is more important than preservation of the original hydrated geometry [62]. UV treatment should be limited to thin or directly exposed hydrogel formats, because effective UV-based microbial control depends on direct irradiation [59].

Overall, sterilization should be considered part of hydrogel formulation and process design, not only a final decontamination step. The relevant question is not which method best preserves the initial hydrogel state, but which post-sterilization hydrogel profile best matches the intended application.

### 2.5. Limitations and Future Perspectives

This study has several limitations. First, the proposed interpretation of sterilization-induced structural changes is based on microbiological outcomes, rheology, dimensional measurements, and swelling behavior. Direct molecular characterization was not performed; therefore, the structural explanations should be interpreted as material-response-based rather than mechanistic conclusions.

Second, the study used one ionically crosslinked alginate formulation as a model system. The observed post-sterilization profiles may differ for hydrogels with other polymer compositions, crosslinking mechanisms, geometries, hydration states, or incorporated bioactive compounds.

Third, storage, transport, handling, and the time between sterilization and analysis may contribute to variability, particularly for highly hydrated samples and externally processed methods such as gamma irradiation and EtO. These factors are relevant because dehydration, water redistribution, and handling-related changes can affect mass, dimensions, swelling, and rheological behavior.

Fourth, microbiological effectiveness was assessed qualitatively as the presence or absence of detectable growth. This approach was suitable for evaluating the tested conditions, but it does not provide detailed inactivation kinetics or log-reduction values.

Future studies should validate the identified post-sterilization profiles in application-specific hydrogel systems and expand this approach to other model systems with different polymer compositions, crosslinking mechanisms, geometries, and hydration states. Molecular characterization, biocompatibility, degradation, drug loading and release, long-term stability, and residual sterilant analysis should be included where relevant. For HHP, optimization of pressure, cycle number, holding time, temperature, hydrogel composition, and microbial target is required. Because these variables are interdependent, machine learning and other data-driven approaches may help define processing windows and predict microbiology–structure trade-offs across hydrogel systems.

## 3. Conclusions

Using ionically crosslinked alginate hydrogels as a model system, this study showed that sterilization is both a microbiological and material-processing step.

Steam sterilization, gamma irradiation, EtO, and selected HHP conditions achieved no detectable microbial growth, whereas UV irradiation was insufficient to eliminate matrix-associated contamination. HHP effectiveness depended on pressure and cycle number, confirming the need for process-specific optimization.

The tested methods produced different post-sterilization hydrogel states, as shown by rheology, dimensional measurements, and swelling behavior. These results demonstrate that microbiological effectiveness alone is not sufficient for selecting a sterilization method for hydrogel systems.

The integrated microbiology–rheology–swelling–geometry assessment showed that no single method simultaneously maximized microbial control and preserved the initial hydrogel state. Therefore, the selection of the sterilization method should be based on the required final hydrogel profile, including microbial safety, mechanical behavior, swelling capacity, dimensional stability, and implementation constraints.

Taken together, sterilization should be considered part of the hydrogel formulation and process design, rather than merely a final decontamination step.

## 4. Materials and Methods

### 4.1. Microbiological Characterization of Samples

In this study, reference bacterial cultures and clinically isolated bacterial strains were used to contaminate experimental samples. Reference cultures of *Escherichia coli* (ATCC 25922), extended-spectrum β-lactamase-producing *Escherichia coli* (ESBL), *Pseudomonas aeruginosa* (ATCC 27853), *Staphylococcus aureus* (ATCC 25923), and methicillin-resistant *Staphylococcus aureus* (MRSA), and *Candida albicans* (ATCC 10231) were used. Both clinical isolates were previously isolated from pus and urine samples and identified using the VITEK 2 system (bioMérieux, Marcy-l’Étoile, France).

Microbial suspensions were prepared in a sterile 0.9% (*w*/*v*) NaCl solution using a densitometer (Biosan, Riga, Latvia) to achieve a 0.5 McFarland standard, corresponding to an approximate cell density of 1.5 × 10^8^ cells/mL.

Sample sterility after sterilization was assessed using classical swab techniques. Sterile cotton swabs were moistened with sterile 0.9% (*w*/*v*) NaCl solution and used to sample both the outer surface and internal cross-sectional surfaces of each hydrogel slice. The entire swab head was applied to obtain material from the sample surfaces. Next, the same cotton swab was used to inoculate the obtained surface swab onto non-selective growth agar plates: trypticase soy agar (Oxoid, Cheshire, UK) for bacteria and Sabouraud dextrose agar (Oxoid, Cheshire, UK) for *C. albicans*. All growth agar plates were incubated in a thermostat (Memmert, Büchenbach, Germany) at 37 °C for 18 h under sterile conditions. Following incubation, microbial growth was assessed using an automatic colony detection system (SCAN-300, Interscience, Saint Nom la Bretèche, France). Microbial growth was assessed qualitatively based on the presence or absence of detectable growth. This approach was considered sufficient for comparative evaluation of sterilization effectiveness across methods. Results were recorded as the presence or absence of detectable microbial growth. Each sample was analyzed in triplicate.

### 4.2. Alginate Sample Preparation

Alginate gels were prepared using a method adapted from Bajpai et al. [63] from a 2% (*w*/*v*) sodium alginate (Sigma-Aldrich, St. Louis, MO, USA, Macklin, Shanghai, China) solution in deionized water, which was degassed prior to crosslinking to remove trapped air and minimize bubble formation within the hydrogel. Fifty milliliters of the solution was transferred into a dialysis tube (Membra-Cel™, MWCO 14,000) (from Dominique DUTSCHER SAS, Bernolsheim, France), which was then immersed in 450 mL of 1.5% (*w*/*v*) calcium chloride (Carl Roth, Karlsruhe, Germany, ≥97%, Ph. Eur., Spain) solution. After 24 h of crosslinking, the tube was removed, and the resulting cylindrical hydrogel was washed with distilled water to remove excess calcium ions. The cylindrical geometry was chosen to minimize variability between samples and preparation batches, ensuring homogeneous, comparable hydrogels and reducing the impact of sample preparation on subsequent analyses.

The hydrogel cylinders were sliced into 1 cm-thick sections for subsequent sterilization and rheological analysis. For each hydrogel section, mass, diameter, and height were measured before and after sterilization to evaluate any sterilization-induced geometric changes.

Swelling behavior was assessed gravimetrically using dried unsterilized and sterilized hydrogel sections. Samples were dried at room temperature (22 ± 1 °C) until constant mass was reached prior to swelling measurements. The dried sections were rehydrated in deionized water and phosphate-buffered saline (PBS pH 7.4) at room temperature under identical conditions. Water uptake was expressed as a percentage increase relative to the dry mass. Room temperature conditions were selected to ensure consistent comparison of sterilization-induced structural changes without introducing additional variables associated with physiological conditions.

### 4.3. Preparation of Contaminated Hydrogel Samples

The contamination strategy was designed to evaluate sterilization under conditions where microorganisms are present not only on the surface but also throughout the hydrogel volume, simulating potential contamination during manufacturing.

**Figure A2:**
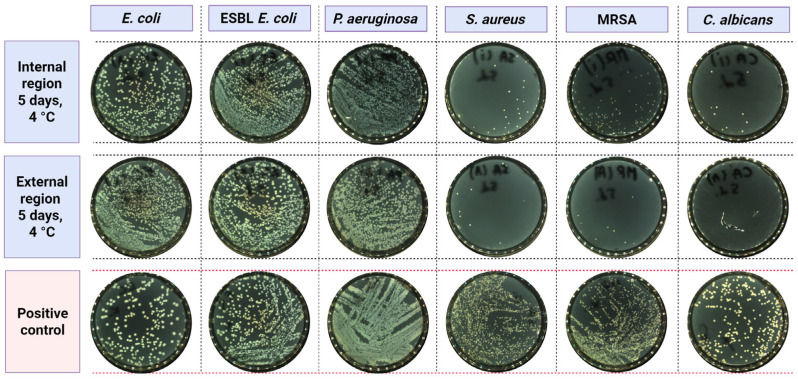
Preliminary viability test for contamination strategy selection. Representative agar plates show microbial recovery from internal and external hydrogel regions after 5 days at 4 °C, with positive controls shown for comparison. Created in BioRender. Brangule, A. (2026) https://BioRender.com/3z4l40r.

The selection of the contamination approach was based on preliminary 5-day microbial stability tests performed at 4 °C for both Gram-negative and Gram-positive microorganisms in Ca^2+^-crosslinked alginate hydrogels. These tests showed that Gram-negative bacteria retained sufficient viability after direct incorporation into the alginate matrix, whereas Gram-positive bacteria and *C. albicans* exhibited a rapid reduction in viability under the same conditions (Appendix A.2, Figure A2).

This was particularly relevant for outsourced sterilization methods such as gamma irradiation and EtO, where transport and processing may require several days. Under such conditions, it becomes difficult to distinguish sterilization-induced inactivation from spontaneous viability loss during storage.

For Gram-negative bacteria (*P. aeruginosa*, *E. coli*, and ESBL-producing *E. coli*), contamination was therefore achieved by direct incorporation into the alginate solution prior to gelation. Briefly, 5 mL of bacterial suspension containing 10^8^ cells/mL was added to 50 mL of 2% (*w*/*v*) sodium alginate solution. The mixture was gently stirred, then transferred into the dialysis tube for ionic crosslinking. This approach enabled homogeneous microbial distribution throughout the hydrogel matrix and allowed sterilization effectiveness to be evaluated for full-volume contamination rather than surface contamination alone.

In contrast, contamination with Gram-positive bacteria (*S. aureus* and MRSA) and *C. albicans* was performed by post-gelation immersion. Pre-formed 1 cm-thick hydrogel sections were immersed in the respective bacterial or fungal suspensions containing 10^8^ cells/mL for 2 h. This approach ensured sufficient and reproducible contamination while avoiding viability loss associated with prolonged direct exposure to the Ca^2+^-crosslinked alginate system. The reduced stability of Gram-positive bacteria under these conditions is consistent with previous reports of increased susceptibility to cell wall stress in Ca^2+^-rich environments.

### 4.4. Sterilization Methods

To evaluate the effects of different sterilization techniques on the alginate hydrogel model system, contaminated and uncontaminated samples were treated with HHP, UV irradiation, EtO sterilization, steam sterilization, and gamma irradiation. Uncontaminated alginate slices were used as a control for rheological and swelling experiments. All experiments were performed in triplicate unless stated otherwise.

To minimize handling-related variability, all hydrogel sections were prepared with the same geometry and handled under identical conditions. Samples were stored in sealed sterile containers at 4 °C. Samples assigned to EtO sterilization returned from external processing within 3–5 days after preparation, whereas gamma-irradiated samples returned within up to 7–10 days after preparation. After sterilization or return from external processing, all samples were analyzed within 24–48 h.

#### 4.4.1. HHP Sterilization

Each 1 cm-thick hydrogel section was placed in a sterile polyethylene bag and sealed. For safety reasons, each sealed bag was placed in a second polyethylene bag and sealed again.

High-pressure treatment was carried out using a pressure vessel (Model PW 100 ES, P/O/WEBER, Remshalden, Germany). Samples were placed into the vertical hydraulic cylinder (7.6 L) filled with hydraulic oil, which served as the pressure-transmitting medium. All experiments were performed at room temperature (22 ± 1 °C). Samples were treated at 100 MPa, 200 MPa, and 300 MPa, with a 10 min holding time and 1 or 4 cycles. The decompression time was 5 min. Pressure, time, and cycle parameters were selected based on our preliminary experiments and previous HHP optimization work.

#### 4.4.2. UV Sterilization

Contaminated alginate hydrogel slices were individually placed on sterile Petri dishes. Each slice was exposed to UV irradiation (Crosslinker CL-50B, 254 nm, UVitec Ltd., Cambridge, UK) for 30 min at a distance of 20 cm using the instrument’s factory-set dose of 0.120 J/cm^2^. After 30 min, the slices were flipped using sterile forceps and irradiated for an additional 30 min. Following irradiation, each sample was carefully transferred using sterile techniques into labeled sterile containers and immediately transported for microbiological analysis.

#### 4.4.3. EtO Sterilization

Hydrogel sections were placed in ethylene oxide-permeable packaging prior to sterilization and processed by a certified external provider (SIA Osmunds, Riga, Latvia) using a validated industrial EtO cycle (total EtO dwell 420 min) in accordance with ISO 11135:2014 [64]. The sterilization process included controlled preconditioning (24 h, RH 80%) and humidification (45 min, RH 94%) steps, with temperatures maintained at 45–50 °C, post EtO evacuation 60 mbar, 9 min).

#### 4.4.4. Steam Sterilization

Single 1 cm thick hydrogel sections were steam-sterilized at 121 °C for 20 min under 179.5 kPa using a tabletop pre-vacuum autoclave (ELARA11, Tuttnauer Europe B.V., Breda, the Netherlands) and cooled prior to use.

#### 4.4.5. Gamma Sterilization

Contaminated hydrogel sections were gamma-sterilized (10, 20, or 40 kGy (±10%)) by a certified external provider (Ionisos Baltics OÜ, Tallinn, Estonia) in accordance with ISO 11137 [65].

### 4.5. Rheology

The rheological properties of the hydrogels before and after sterilization were determined using an Anton Paar MCR 102e rheometer equipped with a parallel-plate geometry (50 mm diameter) and a Julabo Corio CP-200F temperature control system. All measurements were conducted at 37 °C to ensure controlled and consistent measurement conditions. The gap between the plates was set to 5 mm. The LVER was assessed by an amplitude sweep test at a constant oscillation frequency (ω) of 10 rad s^−1^ in the shear strain (γ) range of 10^−3^ × 10^2^%. The storage (G′) and loss (G″) moduli values were extracted from the same amplitude sweep dataset, using values within the LVER.

To verify the reliability and reproducibility of the rheological measurements, method validation was performed using unsterilized control hydrogels. Batch-to-batch variability was evaluated by analyzing hydrogels prepared in three independent batches (*n* = 3) under identical preparation conditions on different days. All measurements were performed in three independent replicates per condition, and results are presented as mean ± SD.

Since the cylindrical hydrogels were formed in a vertical orientation during crosslinking, intra-sample variability was assessed by measuring three distinct positions along the cylinder axis: top, middle, and bottom.

The relative standard deviation (RSD, %) of G′ values within the LVER (first 4–5 points) obtained from the same batch at three distinct positions along the cylinder axis was 9.5%. The RSDs for G″ values and the LVER were 7.5% and 20.7%, respectively. For samples from different batches (top sections only), the RSD of G′ values within the LVER region (first 3–4 points) was 14.7%. The RSDs for G″ values and the LVER were 17.6% and 48.4%, respectively. The relatively higher variability of the LVER boundary reflects the yield behavior’s sensitivity to small structural differences in hydrogel samples.

The full dataset of rheological measurements generated in this study is publicly available in the Zenodo repository listed in the Data Availability Statement.

### 4.6. Statistical Analysis

Statistical analyses were performed using OriginPro 2019b (OriginLab Corporation, Northampton, MA, USA). Quantitative data are presented as mean ± standard deviation (SD) from three independent replicates unless stated otherwise. Statistical comparisons among treatment groups were performed using one-way ANOVA followed by Tukey’s post hoc test where appropriate. Differences were considered statistically significant at *p* < 0.05. LVER was used as a supporting indicator of deformation tolerance because it showed higher replicate-to-replicate variability than G′ and tan δ. Dimensional and swelling responses were interpreted primarily as descriptive bulk-level indicators. Microbiological outcomes were recorded qualitatively and were not subjected to statistical testing.

## Figures and Tables

**Table 1:** Integrated post-sterilization profiles in the alginate hydrogel model system.

Parameter	Steam	Gamma	EtO	HHP	UV
Microbiological outcome	No detectable growth	No detectable growth	No detectable growth	Effective only under selected pressure–cycle conditions	Limited; insufficient for matrix-associated contamination
G′–LVER rheological profile *	Region II: Stiff–high LVER	Region IV: Soft–high LVER	Region IV: Soft–high LVER, highest LVER	Mainly Region III: soft–low LVER	Region I: Stiff–low LVER
Swelling response in PBS after 24 h **	Low; reduced vs. reference	Moderate; dose-dependent decrease	Low; reduced vs. reference	Highest; pressure-dependent increase	Reduced vs. reference
Dimensional response ***	Moderate shrinkage; mass, height, and diameter reduced	Dose-dependent mass loss and shrinkage	Strongest dimensional alteration; marked mass loss, height reduction, and diameter reduction	Pressure-dependent mass and height reduction; limited diameter change	Smallest dimensional change; no major height or diameter change
Cost and implementation burden	Low-cost and widely available; limited by heat and moisture sensitivity [51]	Scalable for packaged products; requires irradiation access, dose validation, and material compatibility assessment [2]	Applicable to heat-sensitive products; requires aeration, residual control, and regulatory validation [52]	Industrially scalable but equipment-intensive; requires high initial investment and pressure–cycle optimization [37,53]	Low-cost surface treatment; limited by penetration depth and shadowing; unsuitable as a standalone method for bulk hydrogels [54,55]
Application-oriented interpretation	Robust or compact hydrogel formats where preservation of original hydrated geometry is not critical [8,51]	Soft terminally sterilized hydrogel formats, provided that dose-dependent material changes are controlled [56]	Flexible or conformable hydrogel formats, provided that residual safety, dimensional stability, and biological performance are validated [57]	Hydrated or thermally sensitive hydrogel systems where high rehydration capacity is acceptable or desirable, after pressure–cycle optimization [58]	Thin or directly exposed hydrogel formats only; not appropriate when bulk sterility is required [8,59]

* G′–LVER regions are defined in Figure 4 and represent relative rheological profiles within this alginate hydrogel model system. ** Swelling response refers to relative differences among dried samples rehydrated in PBS (pH 7.4) for 24 h at 22 ± 1 °C. Categories are interpreted relative to the unsterilized reference hydrogel and comparatively among sterilized groups. *** Dimensional response summarizes post-sterilization changes in mass, height, and diameter after sterilization.

## Data Availability

The data presented in this study are openly available in Zenodo at https://doi.org/10.5281/zenodo.19230556.

## Appendix A

### Appendix A.1

Representative agar plates illustrate microbial recovery after UV irradiation in alginate hydrogels model system. Gram-negative bacteria (*E. coli*, ESBL-producing *E. coli*, and *P. aeruginosa*) were incorporated into the matrix before gelation. *S. aureus*, MRSA, and *C. albicans* were introduced by post-gelation immersion. Microbial recovery was assessed separately from internal and external hydrogel regions. Detectable microbial growth after UV treatment indicates limited microbiological effectiveness under these conditions.

### Appendix A.2

Preliminary 5-day storage tests at 4 °C were conducted to determine the contamination strategy. Microbial recovery was assessed separately for internal and external hydrogel regions. Gram-negative bacteria maintained sufficient recovery after incorporation into the hydrogel matrix, while Gram-positive bacteria and *C. albicans* showed reduced recovery under the same conditions. As a result, Gram-negative bacteria were incorporated before gelation, and Gram-positive bacteria and *C. albicans* were introduced by post-gelation immersion.
